# Tobacco smoking and semen quality in infertile males: a systematic review and meta-analysis

**DOI:** 10.1186/s12889-018-6319-3

**Published:** 2019-01-08

**Authors:** Pravesh Kumar Bundhun, Girish Janoo, Akash Bhurtu, Abhishek Rishikesh Teeluck, Mohammad Zafooruddin Sani Soogund, Manish Pursun, Feng Huang

**Affiliations:** 1 Department of Internal Medicine, the First Affiliated Hospital of Guangxi Medical University, Nanning, Guangxi 530021 People’s Republic of China; 2 Guangxi Medical University, Nanning, Guangxi 530027 People’s Republic of China; 3 Institute of Cardiovascular Diseases and Guangxi Key Laboratory Base of Precision Medicine in Cardio-cerebrovascular Disease Control and Prevention and Guangxi Clinical Research Center for Cardio-cerebrovascular Diseases, the First Affiliated Hospital of Guangxi Medical University, Nanning, 530021 Guangxi China

**Keywords:** Smoking, Infertile men, Semen, Oligozoospermia, Asthenozoospermia, Teratozoospermia, Azoospermia

## Abstract

**Background:**

Nowadays, the total number of couples visiting an infertility clinic is on the rise. Tobacco smoking is considered one of the major factors leading to male infertility. In this study, we aimed to systematically investigate the impact of tobacco smoking on semen quality in infertile male participants.

**Methods:**

Online databases (Cochrane Central database of Randomized Controlled Trials and the databases of MEDLINE and EMBASE respectively) were searched for relevant English publications that satisfied the inclusion and exclusion criteria of this analysis. The clinical endpoints which were assessed included semen parameters (oligozoospermia, asthenozoospermia, teratozoospermia, and azoospermia), morphological defects of spermatozoa and the hormones involved in reproduction. RevMan 5.3 software was used to analyze the data whereby mean difference (MD) and risk ratios (RR) with 95% confidence intervals (CI) were generated to represent the results.

**Results:**

Sixteen studies with a total number of 10,823 infertile male participants (5257 smokers and 5566 non-smokers) were included. Results of this analysis showed oligozoospermia to be significantly higher in smokers (RR: 1.29, 95% CI: 1.05–1.59; *P* = 0.02). Morphological defect of spermatozoa (MD: 2.44, 95% CI: 0.99–3.89; *P* = 0.001) was also significantly higher in smokers whereby significant head (MD: 1.76, 95% CI: 0.32–3.20; P = 0.02), neck (MD: 1.97, 95% CI: 0.75–3.18; *P* = 0.002) and tail (MD: 1.29, 95% CI: 0.35–2.22; *P* = 0.007) defects were observed. However, smoking did not affected the pH (MD: 0.04, 95% CI: [− 0.03–0.11]; *P* = 0.30) and motility (RR: 1.42, 95% CI: 0.97–2.09; *P* = 0.07) of spermatozoa. Additionally, tobacco smoking did not cause any dis-balance in hormones which were involved in reproduction.

**Conclusions:**

In conclusion, with reference to the clinical endpoints which were studied in this analysis, tobacco smoking was associated with a lower sperm count and an increase in the number of morphological defects of spermatozoa. However, the pH and motility of spermatozoa as well as the production of hormones which were involved in reproduction were not affected in this population of infertile males.

## Background

Tobacco smoking among the young generation is becoming worse day by day [1]. The effect of tobacco smoking on lung cancer is already well-known [2]. However, other serious health hazards of smoking have not often well been investigated [3]. Not lately, there has been evidence showing tobacco smoking to have shocking impact on reproductive health irrespective of gender status.

Nowadays, the total number of couples visiting an infertility clinic is on the rise [4]. According to the American Society for Reproductive Medicine, infertility is defined as the inability to achieve pregnancy after a duration period of one year of regular, unprotected sexual intercourse [5].

Tobacco smoking is considered one of the major factors leading to male infertility [6] and recent surveys have demonstrated approximately 120, 000 young men (30 to 50 years old) in the United Kingdom to be impotent due to this bad habit. Male infertility (approximately 50% of the cases of infertility among couples [7]) is gradually leading to depression and other psychological outcomes, and this might be potential signs of serious future consequences.

The impact of tobacco smoking on semen quality has seldom been systematically studied. Therefore, by comparing semen parameters between smokers and non-smokers, we aimed to systematically investigate the impact of tobacco smoking on semen quality in infertile male participants.

## Methods

### Electronic databases and searched strategies

An electronic search was carried out for English language publications through the Cochrane Central database of Randomized Controlled Trials, the databases of MEDLINE (Medical-related publications) and EMBASE respectively. The terms ‘smoking and infertility’, ‘smoking and male infertility’, ‘smoking and semen’, ‘smoking and sperm’, ‘smoking and young males’, ‘infertility and tobacco smoking’, ‘smoking and male health’, ‘smoking, males and impotence’, ‘smoking and infertile men’, ‘smoking and sperm morphology’, ‘smoking and sex’, ‘smoking and sperm count’, ‘smoking and testosterone’, ‘smoking and LH’, ‘smoking and FSH’, ‘smoking and prolactin’ and ‘smoking and sperm motility’ were used to find relevant publications.

To improve this search process, the terms ‘males, men, cigarettes, nicotine, tobacco and non-fertile’ were also included one at a time during the search process. In addition, reference lists of suitable articles were also reviewed for relevant publications.

### Inclusion criteria

Inclusion criteria were based on the following features:

- Studies based strictly on infertile male participants;
- Studies that compared respective semen parameters in smokers versus non-smokers;
- Studies that reported the following endpoints: semen parameters, pH of semen, morphological defects of spermatozoa, types of abnormal structural defects, and hormones which were involved in the functioning of the male reproductive system.

### Exclusion criteria

Exclusion criteria were based on the following features:

- Studies that consisted of fertile/normal male participants;
- Studies that did not compare respective semen parameters in smokers versus non-smokers;
- Studies that did not report the above-mentioned endpoints;
- Duplicated studies.

### Endpoints

Selective endpoints included:

- Oligozoospermia;
- Asthenozoospermia;
- Teratozoospermia;
- Azoospermia;
- Morphological defects of spermatozoa: head, neck or tail defects;
- pH of semen;
- Testosterone level;
- Follicle stimulating hormone (FSH) level;
- Luteinizing hormone (LH) level;
- Prolactin level.

The endpoints have been listed in Table 1.

**Table 1:** Reported endpoints

Study	Selective endpoints reported
Al-Turki2014 ^10^	pH of semen, testosterone level, FSH level, LH level, prolactin level
Al-Turki2016 ^11^	Serum testosterone, semen pH
Anifandis2014 ^12^	Sperm immotility
Caserta2012 ^13^	Oligozoospermia, asthenozoospermia, teratozoospermia, FSH level, LH level
Cui2016 ^14^	Abnormal sperm head, abnormal sperm body, abnormal sperm tail
Gaur2007 ^15^	Oligozoospermia, asthenozoospermia, teratozoospermia
Meri2013 ^16^	Serum pH
Mitra2012 ^17^	Asthenozoospermia (reduced motility), oligozoospermia (low sperm count), teratozoospermia (sperm with abnormal morphology), azoospermia (no sperm count), immotility, sperm head defect, sperm tail defect
Trummer2002 ^18^	Asthenozoospermia, oligozoospermia, teratozoospermia, azoospermia, testosterone, FSH level, LH level, prolactin level
Mostafa2006 ^19^	Amorphous sperm head, pathological sperm midpiece, pathological sperm tails
Osser1992 ^20^	Amorphous sperm head, pathological sperm midpiece, pathological sperm tails
Yu2013 ^21^	Sperm immotility
Zhang2013 ^22^	Semen pH, sperm head defects, sperm neck defect, sperm tail defect
Zhang2015 ^23^	FSH level, LH level, testosterone level
Dikshit1987 ^24^	Immotility of sperms and abnormal morphology
Kunzle2003 ^25^	pH, immotility of sperms and abnormal morphology

Abbreviations: *FSH* follicle stimulating hormones, *LH* luteinizing hormone

### Data extraction and review

The search of studies was carried out with reference to the PRISMA guideline [8]. Six authors (PKB, GJ, AB, ART, MZSS and MP) independently reviewed the articles which were considered relevant to this analysis and data were extracted appropriately. The authors’ names, year of publication, the study design, the endpoints which were reported, the total number of smokers and non-smokers respectively, age of patients, and the total number of events which were reported in each study were carefully extracted.

Any disagreement which was raised was spontaneously resolved by the seventh author (FH).

With the exception of the mean age of the participants, other data at baseline were not included in this analysis for two main reasons:

- Many original studies did not include risk factors and co-morbidities at baseline;
- Baseline features which were reported in certain studies were different from those reported in other studies and a comparison would not have been possible.

### Statistical analysis

The latest version of RevMan software (5.3) was used to analyze the data. This analysis involved both continuous and dichotomous data. Mean and standard deviation (SD) were used during subgroup analysis whereby pooled mean difference (MD) was calculated for the continuous data. For dichotomous data, risk ratios (RR) and 95% confidence intervals (CI) were generated to represent the results.

The statistic Q test and statistic I^2^ test were used to evaluate heterogeneity [9]. During the subgroup analysis, statistical significance was set at a *P* value ≤0.05. A fixed effects model (I^2^ < 50%) or a random effects model (I^2^ > 50%) was used based upon the I^2^ value which was obtained during each subgroup analysis.

Each study was excluded one by one and a new analysis was carried out each time to observe any significant difference compared to the main results which were obtained (sensitivity analysis).

Ethical approval or board review approval was not required for this type of research articles.

## Results

### Search outcomes

Electronic search resulted in a total number of 342 articles. After a proper assessment of the titles and abstracts, we excluded 285 studies. Fifty-seven (57) full-text articles were assessed for eligibility. Among the full-text articles, further studies were eliminated because:

- They included fertile/normal male participants (8);
- They involved infertile couples without specifying the gender (3);
- They reported endpoints which were not considered relevant specifically for this analysis (9);
- They were duplicated studies (21).

**Fig. 1:**
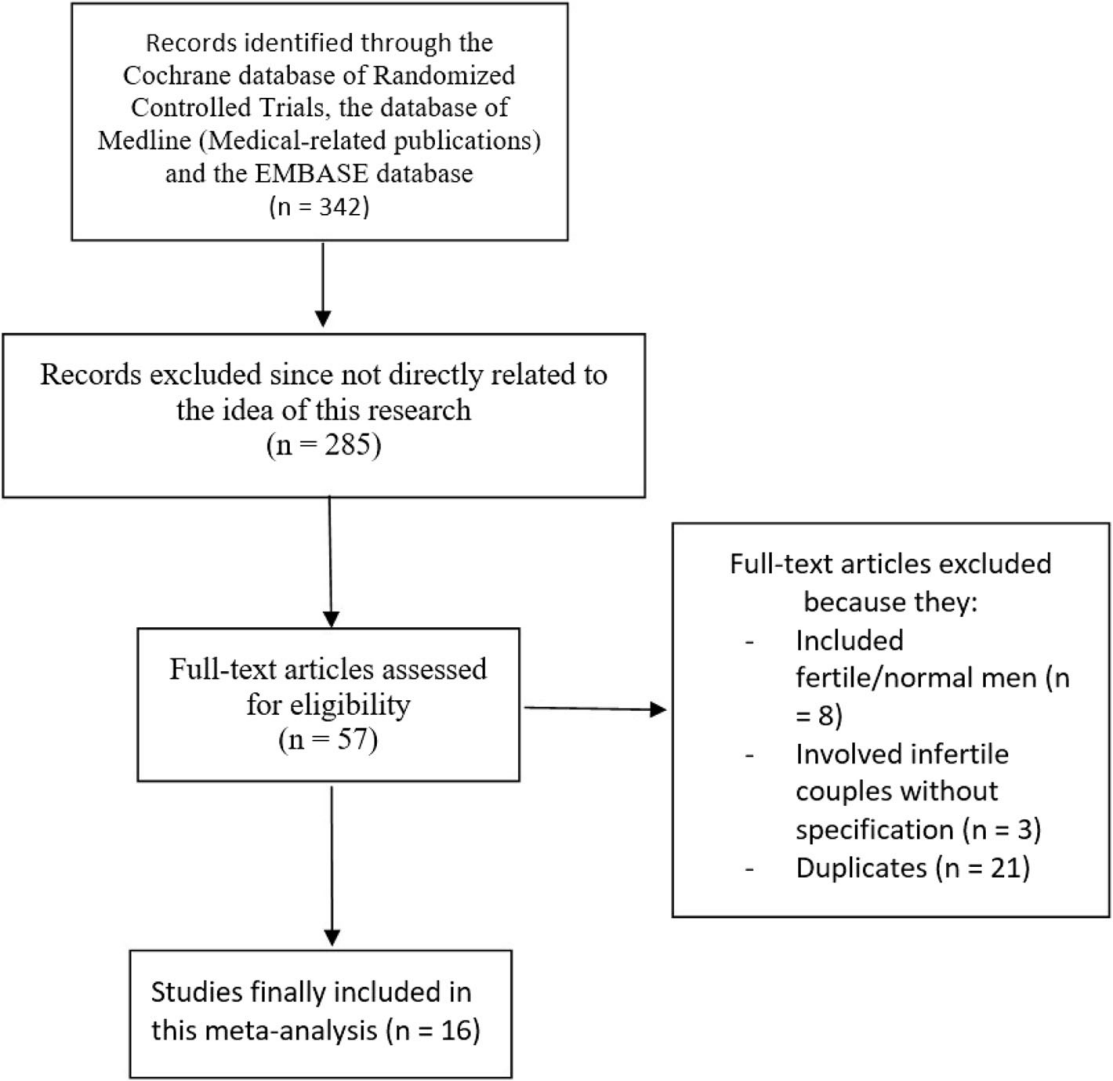
Flow diagram representing the study selection

Finally, 16 studies [10–25] which satisfied all the inclusion and exclusion criteria of this research were included in this analysis (Fig. 1).

### Basic features of the studies which were included in this analysis

A total number of 10,823 infertile male participants (5257 smokers and 5566 non-smokers) were included in this analysis.

The main features of the original studies have been summarized in Table 2.

**Table 2:** General features of the studies

Studies	Study design	Year of patients’ enrollment	No of infertile smokers (*n*)	No of infertile non-smokers (n)	Age (years) S/NS
Al-Turki2014	Retrospective	2010–2012	90	168	34.2/34.1
Al-Turki2016	Retrospective	2008–2013	194	322	34.6/34.3
Anifandis2014	Prospective	–	33	98	37.9/37.1
Caserta2012	Cross sectional	2006–2011	200	448	38.3/38.5
Cui2016	Prospective	2013–2015	920	298	–
Gaur2007	Retrospective	2001–2004	100	100	–
Meri2013	Retrospective	2010–2011	396	564	–
Mitra2012	Cross sectional	–	178	126	40.5/35.0
Trummer2002	Prospective	1993–2000	478	517	31.5/33.4
Mostafa2006	Prospective	–	20	20	–
Osser1992	Retrospective	–	186	164	–
Yu2013	Cross sectional	2011–2012	147	175	35.6/33.6
Zhang2013	Retrospective	2007–2010	737	775	29.6/29.9
Zhang2015	Retrospective	2013–2014	704	372	29.9/30.4
Dikshit1987	Prospective	1985–1986	219	288	26.7/26.5
Kunzle2003	Retrospective	1991–1997	655	1131	32.3/33.2
Total no of patients (n)			5257	5566	

Abbreviations: *S* smokers, *NS* non-smokers

The study design, the participants’ enrollment time periods (1985–2015), the mean age (26.5–40.5 years old), and the total number of smokers (5257 participants) and non-smokers (5566 participants) have been listed in Table 2.

Other characteristics of the participants and the reasons for exclusion have been summarized in Table 3. Majority of the patients did not consume alcohol and the minority who consumed alcohol were only moderate consumers. Participants with varicocele, cryptorchidism, aspermia, chronic diseases, genital infections, genital trauma, chromosomal abnormalities were excluded from this analysis (Table 3).

**Table 3:** Other characteristics and reasons for exclusion of participants

Studies	Type of participants	Alcohol consumption	Reasons for exclusion	Patients identification
Al-Turki2014	Primary and secondary infertility	Alcohol consumption was controlled	Patients with azoospermia	Infertility clinic
Al-Turki2016	Primary and secondary infertility	More than 87% of participants do not consume alcohol	Patients with azoospermia	Infertility clinic
Anifandis2014	Not specified	59.9% participants do not consume alcohol, and 28% were moderate consumers		Infertility clinic
Caserta2012	Primary infertility	Not specified	Patients with azoospermia, orchitis or prostatitis, grade 2 or 3 varicocele, undescended testes or its surgery, altered karyotype	Infertility clinic
Cui2016	Primary infertility	Not specified	Cryptorchidism, varicocele, infections, anti-sperm antibodies, chromosomal abnormalities	Infertility clinic
Gaur2007	Primary infertility	Not specified	Using contraceptive measures, secondary infertility, occupational exposure to chemicals, cryptorchidism, varicocele, chronic illness, leucocytospermia, azoospermia, age > 45 years	Infertility clinic
Meri2013	Not specified	Not specified	Varicocele, undescended testes, small testes, azoospermia, mumps, history of inguinal hernia or scrotal surgery, chronic medical illness	Infertility clinic
Mitra2012	Not specified	Not specified	Pathology of chronic diseases	Infertility clinic
Trummer2002	Not specified	Not specified	Not specified	Infertility clinic
Mostafa2006	Not specified	Not specified	Not specified	Infertility clinic
Osser1992	Not specified	Not specified	Not specified	Infertility clinic
Yu2013	Not specified	Not specified	Unhealthy, varicocele, infection, obstruction of the vas deferens, chromosomal abnormality, azoospermia, severe oligozoospermia, hemospermia, leukospermia, necrozoospermia	Infertility clinic
Zhang2013	Not specified	Not specified	Azoospermia, excessive alcohol intake, hallucinatory drugs, serious systemic disease, abnormality of the external genitalia, known family genital disorders, infection or trauma to genitals	Infertility clinic
Zhang2015	Not specified	Not specified	Not specified	Infertility clinic
Dikshit1987	Screening for idiopathic infertility	No	Past or present systemic disease, alcohol consumption, genital tract disorder, varicocele, genital infection, hormonal abnormalities or treatment, exposure to radiation, drug abuse	Infertility clinic
Kunzle2003	Men attending the andrology laboratory in the context of infertility investigation	Yes	History of orchitis, testicular trauma, sexually transmitted disease, varicocele, inguinal hernia operation and cryptorchism.	Infertility clinic

### Oligozoospermia and teratozoospermia

**Fig. 2:**
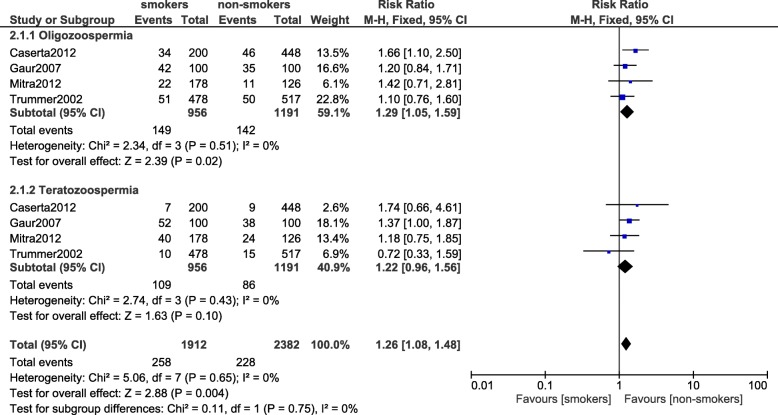
Oligozoospermia and teratozoospermia observed in smoking and non-smoking infertile male participants

Results of this analysis showed oligozoospermia to be significantly higher in smokers (RR: 1.29, 95% CI: 1.05–1.59; *P* = 0.02) whereas teratozoospermia was not significantly different (RR: 1.22, 95% CI: 0.96–1.56; *P* = 0.10) between the smokers and the non-smokers as illustrated in Fig. 2.

### Asthenozoospermia and azoospermia

**Fig. 3:**
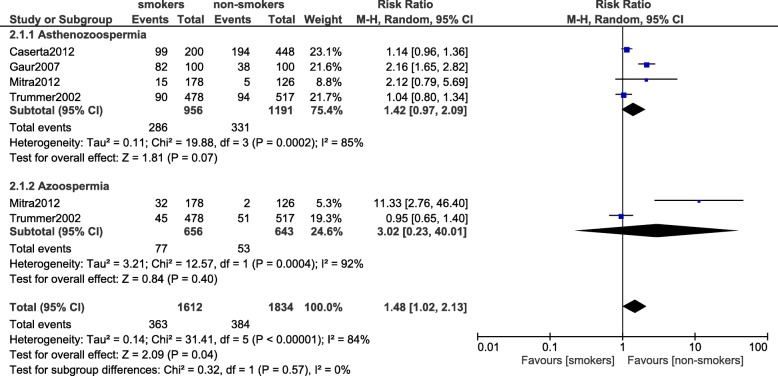
Asthenozoospermia and azoospermia observed in smoking and non-smoking infertile male participants

Asthenozoospermia (RR: 1.42, 95% CI: 0.97–2.09; *P* = 0.07) and azoospermia (RR: 3.02, 95% CI: 0.23–40.01; *P* = 0.40) were not significantly different between the smokers and non-smokers (Fig. 3).

### Impaired motility of spermatozoa and pH of semen (continuous data)

**Fig. 4:**
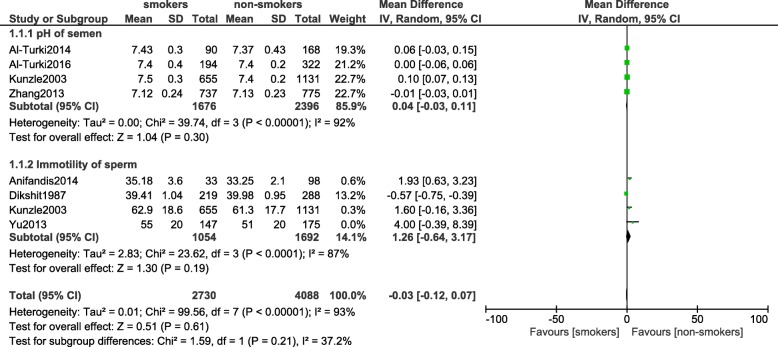
Impaired motility of spermatozoa and pH of semen observed in smoking and non-smoking infertile male participants

The motility of sperms was not impaired between the smokers and non-smokers (MD: 1.26, 95% CI: [− 0.64–3.17]; *P* = 0.19). In addition, pH of semen was also similarly observed (MD: 0.04, 95% CI: [− 0.03–0.11]; *P* = 0.30) [Fig. 4].

### Morphological defects of spermatozoa

**Fig. 5:**
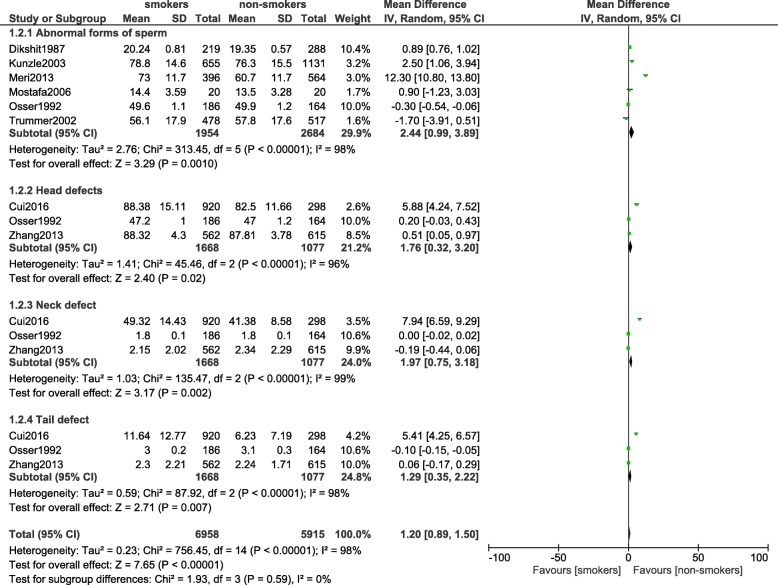
Morphological defects of spermatozoa observed in smoking and non-smoking infertile male participants

There was a significant increase in the morphological defects of spermatozoa (MD: 2.44, 95% CI: [0.99–3.89]; *P* = 0.001) including head (MD: 1.76, 95% CI: 0.32–3.20; *P* = 0.02), neck (MD: 1.97, 95% CI: 0.75–3.18; *P* = 0.002) and tail (MD: 1.29, 95% CI: 0.35–2.22; *P* = 0.007) defects as shown in. Figure 5.

### Hormones which were involved in reproduction

**Fig. 6:**
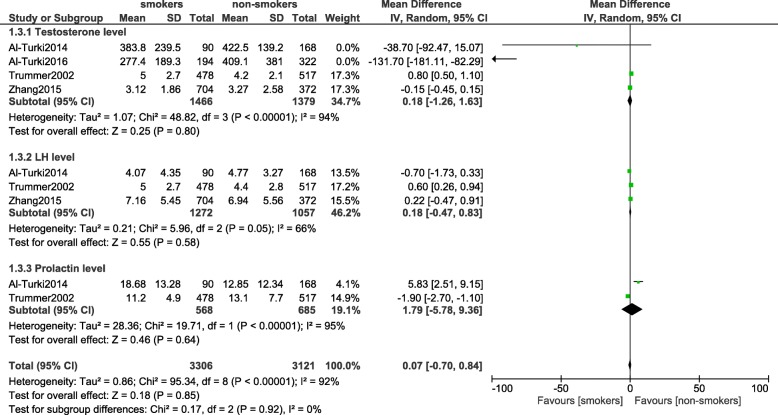
Hormones involved with reproduction observed in smoking and non-smoking infertile male participants (part 1)

**Fig. 7:**
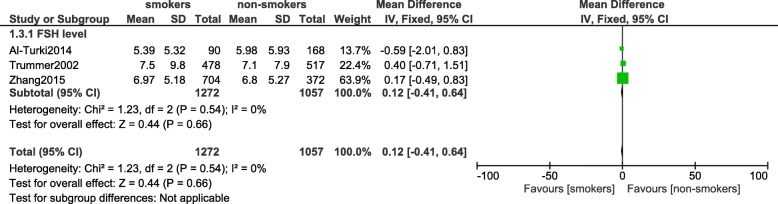
Hormones involved with reproduction observed in smoking and non-smoking infertile male participants (part 2)

This analysis did not show any significant difference in testosterone level (MD: 0.18, 95% CI: -1.26 – 1.63; *P* = 0.80), LH level (MD: 0.18, 95% CI: -0.47 – 0.83; *P* = 0.58) and prolactin level (MD: 1.79, 95% CI: -5.78 – 9.36; *P* = 0.64) between smokers and non-smokers as shown in Fig. 6. FSH level was also not significantly decreased (MD: 0.12, 95% CI: -0.41 – 0.64; *P* = 0.66) [Fig. 7].

Table 4 has summarized the results of this analysis.

**Table 4:** Results of this analysis

Endpoints	No of studies involved (*n*)	RR or MD with 95% CI	P value	I^2^ (%)
Oligozoospermia	4	1.29 [1.05–1.59]	0.02	0
Teratozoospermia	3	1.22 [0.96–1.56]	0.10	0
Asthenozoospermia	4	1.42 [0.97–2.09]	0.07	85
Azoospermia	2	3.02 [0.23–40.01]	0.40	92
pH of semen	4	0.04 [−0.03–0.11]	0.30	92
Impaired motility of sperm (continuous data)	4	1.26 [−0.64–3.17]	0.19	87
Abnormal form of sperm	6	2.44 [0.99–3.89]	0.001	98
Head defects	3	1.76 [0.32–3.20]	0.02	96
Neck defects	3	1.97 [0.75–3.18]	0.002	99
Tail defects	3	1.29 [0.35–2.22]	0.007	98
Testosterone level	4	0.18 [−1.26–1.63]	0.80	94
LH level	3	0.18 [−0.47–0.83]	0.58	66
Prolactin level	2	1.79 [−5.78–9.36]	0.64	95
FSH level	3	0.12 [−0.41–0.64]	0.66	0

Abbreviations: *MD* mean difference, *RR* risk ratio, *CI* confidence intervals, *LH* luteinizing hormone, *FSH* follicle stimulating hormone

Sensitivity analysis showed that in the subgroup analyzing for teratozoospermia, excluding study Mostafa2002 showed a statistically significant result (RR: 1.32, 95% CI: 1.03–1.70; *P* = 0.03). Otherwise, consistent results were obtained throughout all the other subgroups.

## Discussion

As expected, this analysis showed smoking to have a significant impact on the quantity and quality of sperms in the infertile male participants. Tobacco smoking was associated with a lower sperm count and an increase in the number of morphological defects including head, neck and tail defects of spermatozoa. However, the pH and motility of spermatozoa as well as the hormones which were involved in reproduction were not affected in this population of infertile males.

A recent meta-analysis which assessed human semen showed tobacco smoking to have a negative impact on semen parameters [26]. The analysis which consisted of a total number of 5865 fertile and infertile men showed a reduced sperm count and impaired motility in semen samples of these young men. Even though the results which were obtained were almost similar with respect to this current analysis, the other analysis included only studies which were published between the years 2010 to 2015, whereas our current analysis included studies which were published even before the year 2010. Another difference with respect to the current analysis was the fact that there was no language barrier in the other analysis. Moreover, the other analysis also assessed results with reference to the total number of cigarettes which were consumed daily. In contrast to the other analysis, this current meta-analysis assessed specific morphological defects, as well as any dis-balance of the hormones which were involved in reproduction.

Another study evaluating the effect of cigarette smoking on vital seminal parameters which influence fertility showed smoking to cause impaired motility to a higher extent in comparison to the impairment in sperm count [27]. Men with primary infertility aged between 25 to 40 years were included and a follow-up period of less and above 5 years were considered.

A case control study also showed smoking to be associated with a lower semen concentration, impaired motility of spermatozoa and an increased morphology defect [28] in part reflecting the results of this current analysis. Additionally, an article published by the Canadian Society of Clinical Chemists showed that abnormal structural defects of spermatozoa, especially round head defects, were associated with tobacco smoking which might be attributed to increased oxidative stress and insufficient scavenging antioxidant enzymes in the seminal fluids of infertile men [29]. Other studies have shown zinc to contribute to this unwanted mechanism in infertile smokers [30]. Other mechanisms have well been explained in previously published reviews [31, 32].

Briefly, the possible mechanisms which might be involved with the effect of cigarette smoking on semen parameters are: toxic contents found in cigarette smoking might have harmful effects on male germ cells and their developmental processes [33]. Negative effects of nicotine on semen parameters have also previously been reported [34]. Other possible mechanisms might be related to the negative impact of smoking on the 8 nAChR subunits found in human spermatozoa, resulting in smoking-related sperm damage [35]. In addition, different proteins (Aldoa, ATP5a1, Gpx4, Cs) expressed in sperms were significantly altered in smokers [36]. Cigarette smoking was found to also affect Ca^2+^- ATPase activity of the spermatozoa as well [37].

However, even though clinical research has shown smoking to have an adverse effect on the progressive sperm motility irrespective of the total number of cigarettes smoked daily [38, 39], other studies showed no relationship between smoking and male infertility [40].

This current analysis showed no significant influence of smoking on testosterone, prolactin, FSH and LH levels. To support this point, Wang et al. showed smoking not to be an independent predictor of sex-hormone binding globulin even though a relation or linked was observed between increasing packets of cigarette and sex-hormone binding globulin [41]. Similarly, another study conducted in Taiwan showed no significant difference in LH and FSH levels between smokers and non-smokers [42] showing smoking to have a much higher impact on semen compared to the production of hormones which were involved in the functioning of the male reproductive system.

Several alternative methods to stop smoking have been suggested [43–46]. However, apart from smoking, other factors such as regular heavy alcohol consumption [47], certain medications, co-morbidities, autoimmune diseases and other environmental factors might also contribute to abnormalities in semen parameters, morphology and impaired motility and should further be investigated [48].

This interesting research should inspire other scientists to investigate more about the mechanisms, the factors associated with a poor semen quality in smokers; in order for proper actions to be taken in a timely manner to reduce this serious dilemma faced by several young men and couples in our society.

This meta-analysis should be considered new for the following reasons: it is among the only few meta-analyses to systematically show the impact of smoking on the quality of semen in infertile males. This article might be considered new on the basis of the total number of participants and the number of different endpoints which were analyzed in one particular study.

### Limitations

Limitations might be the fact that a high level of heterogeneity was observed among several of the subgroups analyzing the different endpoints. This could be due to the inclusion of observational data. In addition, several endpoints were analyzed only using a small number of studies. Factors such as alcohol consumption could have had an influence on the main results. Moreover, the infertility duration, and other associated factors such as genital infections, varicocele, environmental factors were not clearly reported in several studies.

## Conclusions

In conclusion, with reference to the clinical endpoints which were studied in this analysis, tobacco smoking was associated with a lower sperm count and an increase in the number of morphological defects of spermatozoa. However, the pH and motility of spermatozoa as well as the production of hormones which were involved in reproduction were not affected in this population of infertile males.

## Abbreviations

CI: confidence intervals
FSH: follicle stimulating hormones
LH: luteinizing hormone
RR: risk ratios
